# Short-term effect of acute and repeated urinary bladder inflammation on thigmotactic behaviour in the laboratory rat

**DOI:** 10.12688/f1000research.6255.1

**Published:** 2015-05-08

**Authors:** Rosemary H Morland, Amparo Novejarque, Wenlong Huang, Rachel Wodarski, Franziska Denk, John D Dawes, Tim Pheby, Stephen B McMahon, Andrew SC Rice

**Affiliations:** 1 Pain Research Group, Department of Surgery and Cancer, Faculty of Medicine, Imperial College , London, UK; 2 Wolfson Centre for Age Related Disease, King's College London, London, UK; 3 The Nuffield Department of Clinical Neurosciences, Medical Sciences Division, University of Oxford, Oxford, UK

**Keywords:** Pain, Inflammation, Open Field, Cytokines, Amygdala, Behaviour, c-Fos

## Abstract

Understanding the non-sensory components of the pain experience is crucial to developing effective treatments for pain conditions. Chronic pain is associated with increased incidence of anxio-depressive disorders, and patients often report feelings of vulnerability which can decrease quality of life. In animal models of pain, observation of behaviours such as thigmotaxis can be used to detect such affective disturbances by exploiting the influence of nociceptive stimuli on the innate behavioural conflict between exploration of a novel space and predator avoidance behaviour. This study investigates whether acute and repeated bladder inflammation in adult female Wistar rats increases thigmotactic behaviour in the open field paradigm, and aims to determine whether this correlates with activation in the central amygdala, as measured by c-Fos immunoreactivity. Additionally, up-regulation of inflammatory mediators in the urinary bladder was measured using RT-qPCR array featuring 92 transcripts to examine how local mediators change under experimental conditions. We found acute but not repeated turpentine inflammation of the bladder increased thigmotactic behaviour (decreased frequency of entry to the inner zone) in the open field paradigm, a result that was also observed in the catheter-only instrumentation group. Decreases in locomotor activity were also observed in both models in turpentine and instrumentation groups. No differences were observed in c-Fos activation, although a general increased in activation along the rostro-caudal axis was seen. Inflammatory mediator up-regulation was greatest following acute inflammation, with CCL12, CCL7, and IL-1β significantly up-regulated in both conditions when compared to naïve tissue. These results suggest that acute catheterisation, with or without turpentine inflammation, induces affective alterations detectable in the open field paradigm accompanied by up-regulation of multiple inflammatory mediators.

## 1. Introduction

Pain is a complex experience, dependent on the interplay between sensory aspects and centrally-mediated affective, motivational, cognitive, and behavioural elements. The current translational difficulties in developing effective pain relief are associated in part with a pre-clinical focus on positive sensory signs, whereas the clinical burden of chronic pain comes largely from negative behavioural responses, such as avoiding behaviours perceived as exacerbating, and a general decrease in quality of life as a result of unpredictable spontaneous pain ( Hummel *et al.*, 2008; Suskind *et al.*, 2013). This study uses an animal model to increase our understanding of the non-sensory components of pain by investigating whether acute and repeated visceral inflammation influence behaviour, if alterations are associated with activational changes in central brain areas implicated in generation of affective behavioural responses, and investigate the local changes in cytokine levels twenty-four hours after inflammation.

Visceral pain affects 16–25% of the general population ( Collett, 2013), and is experienced by virtually all at some point, if only transiently. Despite this, visceral pain is inherently difficult to study in animal models, due to sparse innervation and referred pain complicating precise location of origin. This is reflected in the literature. A PubMed search conducted on the 22 ^nd^ June 2014 (search terms: (pain) AND (neuropathic) vs. (pain) AND (visceral)) revealed 12,938 results for pain with a neuropathic element, compared to 5035 for pain with a visceral component. Of these, 510 neuropathic studies were conducted *in vivo*, compared to only 40 for visceral (additional search term: AND ( *in vivo*)). None of these *in vivo* visceral pain publications involved the open field paradigm (additional search paradigm: AND (open field)), which was one of the key reasons for this study. We chose the turpentine model of visceral inflammation due to its simplicity of induction and demonstrated phenotype of viscero-visceral hyper-reflexia and referred hyperalgesia ( Jaggar *et al.*, 1999), that is reversible with analgesics ( Farquhar-Smith & Rice, 2001; Jaggar *et al.*, 1998; Rice, 1995).

To detect negative behavioural responses in animals, complex behavioural outcomes are commonly used. Thigmotaxis is a behaviour characterised by a preference for movement along a surface (i.e. “wall-hugging”), which shows an inverse relationship with exploration of novel areas in rats. It is hypothesised as related to risk assessment and predator avoidance, with the presence of spontaneous or ongoing pain decreasing potentially risky behaviours, such as exploration. The open field paradigm is capable of detecting these subtle behavioural differences in experimental models of pain with varying aetiologies including antiretroviral therapy-induced neuropathy ( Huang *et al.*, 2013; Wallace *et al.*, 2007; Wallace *et al.*, 2008), chemotherapy-induced neuropathy ( Barzegar-Fallah *et al.*, 2014), spinal nerve transection ( Blackbeard *et al.*, 2012), spinal nerve ligation ( Ewan & Martin 2014; Kontinen *et al.*, 1999; Suzuki *et al.*, 2007), chronic constriction injury ( Grégoire *et al.*, 2012), and post-traumatic peripheral nerve trauma ( Medico *et al.*, 2004).

Pain acts on multiple levels within the nervous system: sensory signals from the periphery are received centrally and interpreted in relation to previous experience and current circumstances to produce a behavioural response. The different subdivisions of the amygdala receive sensory input from areas such as the thalamus and spinal cord (capsulo-lateral portion of the central amygdala, via the lateral and basolateral amygdalae), and provides output via the medial nucleus of the central amygdala to the pre-frontal cortex and hypothalamus. This pattern of connectivity suggests involvement in the assessment and generation of emotional associations that form an integral part of the pain experience ( Neugebauer *et al.*, 2009). Studies have shown pain-associated increases in amygdala activity both clinically and pre-clinically in pancreatitis ( Frøkjær *et al.*, 2011), cluster headache ( Seifert *et al.*, 2011), back pain, arthritis ( Baliki *et al.*, 2008), fibromyalgia ( Harris *et al.*, 2009), and menstrual pain ( Tu *et al.*, 2010). In this study, we look at c-Fos immunoreactivity (a marker of persistent neural activation; Barth, 2007) in the amygdala with respect to open field activity, considering whether the presence of acute or persistent visceral inflammation influences c-Fos expression.

Cytokines and associated inflammatory mediators are up-regulated in pain conditions with an inflammatory component ( Calvo *et al.*, 2012; Cheppudira *et al.*, 2009; Girard *et al.*, 2011; Johansson *et al.*, 2003; Nowak *et al.*, 2012; Quan-Xin *et al.*, 2012; Skurlova *et al.*, 2010; Zhang *et al.*, 2013). In particular, Dawes and co-workers demonstrated commonality between cytokines up-regulated following UVB-irradiation in clinical and pre-clinical models, showing the pro-inflammatory cytokine CXCL5 is up-regulated and capable of producing hypersensitivity in otherwise naïve rats ( Dawes *et al.*, 2011). In this study, we hope to show the profile of inflammatory mediators up-regulated following bladder inflammation and investigate whether this profile changes with repeated inflammation.

This study aims to investigate the acute and medium-term effects of visceral inflammation on thigmotactic behaviour, correlating differences in behaviour with both central activation (c-Fos immunoreactivity in the amygdala), and peripheral levels of inflammatory cytokines to further understand the behavioural implications of visceral inflammation.

## 2. Materials and methods

### 2.1 Ethical statement

All animal experiments conformed to British Home Office Regulations (Animals (Scientific Procedures) Act 1986 Amendment Regulations 2012 (SI 2012/3039) and were performed under the authority of United Kingdom Home Office Project Licence 70/7162, adhering to the International Association for the Study of Pain (IASP) guidelines for *in vivo* research ( Zimmermann, 1983; and http://www.iasp-pain.org). Experiments were designed according to Good Laboratory Practice standards ( Macleod *et al.*, 2009) and reported in accordance with the ARRIVE Guidelines ( Kilkenny *et al.*, 2010; Rice *et al.*, 2013). Table 1 shows the major domains of good laboratory practice followed.

**Table 1.** Major Domains of Good Laboratory Practice (after Macleod *et al.*, 2009).

Characteristic	Description of procedures
Sample size calculation	Group size was determined by sample size estimation for each experiment by SigmaStat software, version 3.5 (ANOVA sample size, desired power = 0.8, α = 0.05). Effect sizes for estimation were derived from previous studies in our group.
Inclusion and exclusion criteria	Animals which died prior to/during model induction were excluded from further study. There were no exclusion criteria for the open field study; animals were excluded from c-Fos analysis if >90min elapsed between open field and perfusion, or if sections were badly damaged.
Randomization	Animals were randomised to model group (naïve, VC, TC) by cage using a pseudorandom ABCBCACAB labelling system.
Allocation concealment	The person creating the model (i.e. instillation of turpentine or olive oil) was theoretically unaware of the allocation to treatment group, but due to the pungent odour of turpentine, this was difficult to maintain. Procedures were still followed. This was achieved by the blinding procedure described below, as well as masking cage labels or turning around the cages before each behavioural assessment session.
Reporting of animals excluded from analysis	All animals excluded are reported in Figure 1.
Blinded measurement, assessment, and analysis of outcome	Codes were assigned to different treatments by an independent person and kept in a sealed envelope. The codes were not broken until the analysis had been completed. The experimenter was blinded to the experimental group to which an animal was randomized. In addition, open field videos were renamed by an independent person before analysing, and immunohistochemistry sections were identified by animal not group code.

### 2.2 Experimental animals

Female Wistar rats (Charles River, UK, RRID:RGD_737929), weighing 180–200 g on arrival, were housed in groups of 3–4 in individually ventilated cages with free access to food (RM1 (P), Special Diet Services, UK) and tap water. Animals were maintained under a 12 hour light cycle (07:00–19:00) in temperature and humidity-controlled conditions (25°C, 30%, both ± 5). Cages were cleaned weekly on a Tuesday (p.m.), and housed in a room containing mice and rats of both sexes. Animals were habituated to the holding room for a minimum 48 hours after delivery from the main campus facilities.

### 2.3 Study design

Sample size calculations used data from studies looking at the effect of neuropathic injury (spinal nerve transection) on frequency of inner zone entry in the open field ( Morland *et al.*, 2015 manuscript in preparation). Using a power (1-β) of 0.08 and alpha (α) value of 0.05, a sample size of 8 was calculated as sufficient to detect alterations in thigmotaxis.

**Figure 1.**
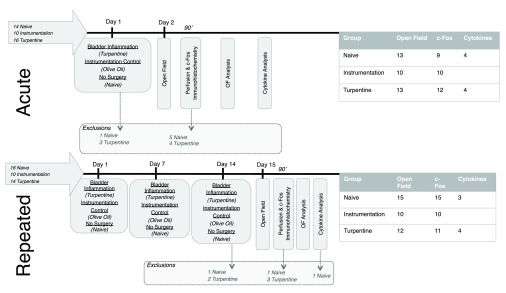
Experimental design for both acute and repeated models. The arrows on the right show the total number of animals used, with exclusions at each timepoint and final group sizes indicated in the central and right of the diagram respectively. Animals were lost during surgery due to complications associated with anaesthesia, and excluded from immunohistochemical analysis if hemispheric differentiation was not possible.

There were three experimental groups: naïve, instrumentation (anaesthesia with catheterization and instillation of 0.5ml 100% olive oil), and turpentine (anaesthesia with catheterization, and inhalation anesthetic instillation of 0.5ml 50% turpentine). Full details of group sizes and exclusions are given in Figure 1. Female animals were selected for ease of catheterization.

All behavioural experiments were conducted during the light phase (09:00–18:00) in a dedicated behavioural laboratory, with surgical procedures carried out in a separate but adjacent surgical room. *In vivo* studies were conducted in batches of 2–3 animals per group (n=6–9/batch) due to capacity and protocol constraints. Each animal was treated as a single experimental unit, with immunohistochemical analysis conducted using average values from a number of sequential sections from a single animal, as summarized in Table 2.

**Table 2.** Average number of sections analysed per animal during immunohistochemical analysis.

		Mean	Min	Max
Acute	Naïve	5.10	4	7
	Instrumentation	4.88	4	7
	Turpentine	4.46	3	6
Repeated	Naïve	2.87	1	5
	Instrumentation	3.90	2	6
	Turpentine	3.83	1	7

### 2.4 Model induction

Under isoflurane anaesthesia, (1.5–3% in 2 L/min O _2_), bladder inflammation was induced as described by McMahon & Abel (1987). Briefly, a transurethral catheter (⌀ 1.02mm; Portex, UK) was introduced into the bladder, and position verified by applying gentle abdominal pressure to empty the bladder, before instillation of 0.5 ml olive oil or turpentine (50% in olive oil). The instillation was maintained under anaesthesia for 2 hours before removal of the catheter. The animal was allowed to recover in a separate area before returning to the home cage.

Based on the above protocol, a model of persistent bladder inflammation was developed, involving three instillations, each one week apart. For both studies, surgery was conducted during the light phase and timed to ensure exactly 24 hours between instillation and exposure to the open field.

### 2.5 Cytokine activation

Tissue from whole bladders snap frozen during the saline phase of perfusion was used for RNA extraction. Briefly, samples were homogenized and total RNA obtained using a “hybrid” method of phenol extraction (Trizol, Invitrogen, USA) and column purification (RNeasy, Qiagen, USA). All samples were deoxyribonuclease (DNase, Qiagen, USA) treated to avoid genomic contamination. Purity and integrity confirmed with an RNA 6000 Nano Chip (Agilent, USA). Complementary DNA (cDNA) was synthesized from RNA using a SuperScript II reverse transcriptase kit (Invitrogen, USA).

Custom-made Taqman array cards (as described by Dawes *et al.*, 2011), featuring four sets of 92 different primer pairs, and four house-keeping genes: glyceraldehyde-3-phosphate dehydrogenase (GAPDH), 18S ribosomal protein, β-actin, and β2-microtubulin. Inflammatory mediators were categorized into cytokines, chemokines, growth factors, enzymes, and ‘other’ ( Table S1). Each cDNA sample was diluted with PCR-grade water and added in a 1:1 ratio to Taqman Universal master mix to produce a final concentration of 1 ng/µl cDNA. Samples were loaded into the appropriate ports (1 µl/well) according to manufacturer guidelines. Cards were placed into a 7900HT Fast Real-Time PCR system (Applied Biosystems, USA), and subjected to 40 cycles of amplification. Transcript expression was measured with the ΔΔ *C _t_* (cycling time) method normalised to the geometric mean of the four housekeeping genes using the R package NormqPCR ( https://r-forge.r-project.org/projects/qpcr/). Relative changes in transcript levels are presented as fold change (FC). When transcript numbers were undetermined for a given detector in <50% of samples, the average *C _t_* value was calculated with the remaining data values. If transcript numbers were undetermined in >50% of transcripts for a given sample, a default *C _t_* of 38 was assigned. If these conditions coincided, no FC value was calculated. Difference in gene expression were detected using the significance analysis of microarray (SAM) technique, involving calculation of false discovery rates (FDR), represented as a *q* statistic ( Lin *et al.*, 2008; Xiao *et al.*, 2002).

### 2.6 Thigmotaxis in the open field

Humidity and temperature were maintained at 25°C and 30% humidity respectively. Light levels outside the isolation chamber during open field paradigms ranged from 70–300 lux. As rodents have a greater auditory range than humans, ultrasonic sound recordings were taken using a Mini-3 Bat Detector (Ultra Sound Advice, UK) to determine the background levels of high frequency sound generated by equipment. Fluorescent lighting and computer equipment emitted signals within the 20–50 kHz range - this equipment was switched on for the duration of each experiment and animals were allowed to acclimatise to the testing room for 30 min prior to testing.

***Thigmotaxis.*** The open field paradigm was used to assess thigmotaxis. The open field arena (black, 100 cm ^2^) was enclosed in an isolation chamber (115 × 115 × 255 cm) to minimise environmental interference. Light levels within the open field were set at 12 lux (measured in the centre of the arena). Animals were introduced into the near corner of the arena, facing the centre of the arena, and allowed to explore for 15 min. The arena was cleaned with 0.02% Distel (formerly Trigene, Tristel Solutions Ltd., UK) between trials. Behaviour was captured by high sensitivity camera (VCB 3372; Sanyo, Japan), and analysed using Ethovision XT 10.1 (Tracksys, UK (for Noldus, the Netherlands), RRID:rid_000100). The primary pre-determined outcome measure was frequency of entry into a virtual central zone (40 cm ^2^). Secondary outcome measures were duration in the central zone, and rearing. Total distance travelled was used as a measure of general locomotor activity. Rearing was defined as both forelimbs elevated, either against a wall, or freestanding, and measured by a trained observer watching at 4 × playback speed.

### 2.7 Amygdala activation

***Histological studies.*** To capture peak c-Fos activation in response to the open field, fixation perfusion took place within 90–120min of open field exposure. Bladders were extracted and snap frozen in LN _2_ during the saline phase of perfusion fixation.

Animals were humanely killed with pentobarbital and transcardially perfused with 0.9% heparinized saline followed by 4% paraformaldehyde. The brain was removed and post-fixed in 4% paraformaldehyde for 6 hours and cryo-protected in 30% sucrose for at least 3 days prior to sectioning (50 µm) on a freezing microtome (model no. HM450, Thermo Scientific, USA). Sections were washed in phosphate buffered saline (PBS; 1(NaH _2_PO _4_.2H _2_O):9(Na _2_HPO _4_.12H _2_O):7(NaCl) in dH _2_O), quenched with 0.03% H _2_O _2_ (Sigma-Aldrich, UK), washed again in PBS, blocked for one hour in 5% normal goat serum (NGS, Millipore, UK; PBS with 0.3% TX (Triton X-100, BDH, UK)), before incubation overnight at 4°C with 1:20,000 polyclonal rabbit IgG anti-c-Fos (Santa Cruz Biotechnology Cat# sc-52 RRID:AB_2106783; 0.3% PBS-TX, 2% NGS). The following day, sections were washed in PBS and incubated for two hours at room temperature (20°C) with 1:250 Biotin-SP-conjugated Affinipure goat anti-rabbit IgG (F(ab’)2 fragment specific; Jackson ImmunoResearch, USA, Cat# 111-036-006 RRID:AB_2313586; 0.3% PBS-TX, 2% NGS). Staining was visualised using a Vectastain ABC kit (avidin-biotin-peroxidase complex, VectorLabs, UK) and DAB (3, 3′-Diaminobenzidine) with nickel salt intensification (VectorLabs, UK). Sections were mounted, counterstained with toluidine blue to enhance cyto-architecture, and cover-slipped using DePex mounting media (VWR, UK) prior to image capture.

***Image quantification.*** The bregma position of each mounted section was determined with reference to Paxinos & Watson (6 ^th^ Edition, 2007), and only those between -1.44mm and -3.36mm (range of the central amygdala) were captured using a Leica DM R light microscope (Leica Microsystems, Germany). Image analysis was conducted using Photoshop CS5 (Adobe, USA), and the mean number of positively stained cells per mm ^2^ calculated for each subdivision of the central amygdala (medial, lateral, and capsular), with lateralization and rostro-caudal axis position noted. A positively stained cell was defined as having a clearly defined dark rounded nucleus (blue/black) - sections without c-Fos positive cells in areas out-with the central amygdala were excluded. Image analysis was conducted blind, using individual animal identifiers rather than group codes until completion of analysis.

### 2.8 Statistical analysis

Thigmotaxis was analysed using 1-way ANOVA, with Kruskal-Wallis utilised for data that was not normally distributed. c-Fos data was analysed using 1-, 2-, and 3-way ANOVA taking account of hemispheric, sub-nuclear, and rostro-caudal designations of the central amygdala. Where overall ANOVA detected significant differences, multiple comparisons procedures were used - Holm-Sidak for normally distributed data, and Dunn’s multiple comparison for non-parametric tests. Correlations were assessed using Pearson correlation coefficients. Inflammatory mediator analysis was conducted using Significance Analysis of Microarrays (SAM), and the false discovery method (FDR), which take into account dependence between transcripts and uses non-parametric techniques. Significance was taken as q=0%.

Summary statistics are expressed as mean (standard deviation; SD) when data was normally distributed, or median (interquartile range; IQR) when data failed normality testing. Significance was taken at p<0.05.

All statistical tests were performed, sample sizes calculated, and appropriate graphs generated using OriginPro v9.1 (OriginLab, USA) and SigmaPlot v10 (Systat Software Inc., USA; RRID:SciRes_000184).

## 3. Results

**Dataset 1**. *Figshare:* Cytokine q-RT-PCR, c-Fos immunoreactivity and open field behaviour data in rats following bladder inflammation. doi: 10.6084/m9.figshare.1394861

### Exclusions

See Figure 1 for a summary of exclusions and group sizes used. Bladder inflammation as a model of visceral inflammation was generally well tolerated; the main cause of mortality was surgical complications and related anaesthesia. In the repeated model, all deaths occurred during/following the final inflammation session. Following recovery from anaesthesia and return to the home cage, animals were alert and outwardly indistinguishable.

### 3.1 Inflammatory mediator mRNA levels

***3.1.1 Acute bladder inflammation.*** A total of 81/92 cytokine transcripts were analysed for magnitude and significance of transcript up-regulation following acute bladder inflammation. 11 markers (CCL1, CCL28, CTLA-8, CXCL17, IFNγ, IL-2, IL-3, IL-4, IL-9, IL-13, IL-27) were excluded from the acute inflammation model due to high cycle time (>38), indicative of low levels or issues in detection. The top ten up-regulated transcripts are shown in Table 3. Figure 1 shows the rank FC for all markers analysed. Significance analysis of microarrays (SAM) was used to identify 25 mRNAs that were significantly different in the turpentine group compared to naive (FC >1.5, Δ = 1.61, FDR=0%). Of these, 13 were classified as chemokines, 9 as cytokines, 2 as enzymes, 2 as growth factors, and 1 as ‘other’, as seen in Table 4.

**Table 3.** Top 10 up-regulated transcripts in bladder tissue from turpentine group, ranked by mRNA fold change normalised to naive values. Data presented as mean fold change (95% confidence interval), n=3–4 animals/group.

	Acute Bladder Inflammation			Repeated Bladder Inflammation		
Rank	Gene	Fold Change	95% Confidence Interval	Gene	Fold Change	95% Confidence Interval
1	NOS2	159110.28	-127366.6–445587.2	CCL12	169.65	67.05–272.25
2	PROK2	11060.01	-12741.5–34861.5	CTLA-8	75.098	-56.21–206.4
3	CXCL2	3679.32	-4618.0–11976.7	CXCL17	71.2	-22.64–165.03
4	CCL3	2541.99	-1417.4–6501.3	CXCL11	53.27	-71.05–177.6
5	IL-1α	1827.32	-2717.6–6372.2	NOS2	14.2	-9.46–37.87
6	IL-1β	403.78	-328.2–1135.7	BDNF	11.9	-2.06–25.86
7	CSF3	350.52	-269.9–971.0	CXCL2	9.94	-9.78–29.65
8	CXCL3	254.33	-131.8–640.5	PROK2	9.12	-10.24–28.49
9	IL-10	222.75	-399.6–845.1	IL-21	8.88	-17.20–34.96
10	IL-6	153.21	-42.8–349.3	IL-1β	8.63	4.62–12.65

**Table 4.** Significance analysis of qRT-PCR array (SAM) results for acute and repeated bladder inflammation. Significant fold difference in acute model ^a^, repeated model ^b^, or both ^c^; Significance level, FDR q=0% denoted by bold text.

		Acute			Repeated		
		FC	Score (d)	q-value(%)	FC	Score (d)	q-value (%)
Chemokines	CCL2 ^a^	**43.47**	4.63	0.00	3.36	1.43	8.44
	CCL3 ^a^	**1656.98**	8.89	0.00	*transcript excluded*		
	CCL4 ^a^	**95.66**	2.55	0.00	6.74	1.13	8.44
	CCL6 ^a^	**14.34**	2.52	0.00	3.98	1.90	5.52
	CCL7 ^c^	**17.96**	2.37	0.00	**5.44**	3.47	0.00
	CCL12 ^c^	**149.46**	3.70	0.00	**120.42**	8.28	0.00
	CCL20 ^a^	**66.21**	3.69	0.00	9.71	1.40	8.44
	CXCL1 ^a^	**218.46**	2.71	0.00	3.42	1.21	8.44
	CXCL2 ^a^	**9412.93**	5.23	0.00	5.63	1.22	8.44
	CXCL3 ^a^	**75.99**	2.61	0.00	*transcript excluded*		
	CXCL6 ^a^	**106.35**	4.74	0.00	5.24	1.17	8.44
	CXCL17 ^b^	*transcript excluded*			**34.53**	4.15	0.00
	XCL1 ^a^	0.01	-4.93	0.00	2.38	0.45	15.95
Cytokines	CSF2 ^a^	**91.78**	5.87	0.00	2.20	0.57	15.95
	CSF3 ^a^	**1355.83**	4.75	0.00	5.23	0.87	10.67
	IL-1α ^a^	**2857.26**	4.63	0.00	5.46	1.60	5.52
	IL-1β ^c^	**272.74**	5.88	0.00	**7.15**	2.68	0.00
	IL-6 ^a^	**522.18**	4.78	0.00	4.57	1.11	8.44
	IL-10 ^a^	**334.63**	3.67	0.00	2.83	0.48	15.95
	IL-24 ^a^	**233.58**	4.22	0.00	0.11	-0.98	21.98
	IL-35 / Ebi3 ^a^	**45.77**	2.46	0.00	3.15	1.10	8.44
	Tnf ^a^	**20.44**	3.20	0.00	2.42	1.13	8.44
Enzymes	Nos2 ^a^	**81737.07**	11.43	0.00	13.87	1.98	5.52
	COX-2 / Ptgs2 ^a^	**8.42**	2.66	0.00	0.96	-0.07	21.98
Growth Factors	Artn ^a^	**0.37**	-2.46	2.26	0.39	-1.61	20.25
	Ereg ^a^	**140.20**	3.15	0.00	24.38	1.91	5.52
Other	PROK2 ^a^	**4443.04**	7.25	0.00	8.52	1.34	8.44

**Figure 2.**
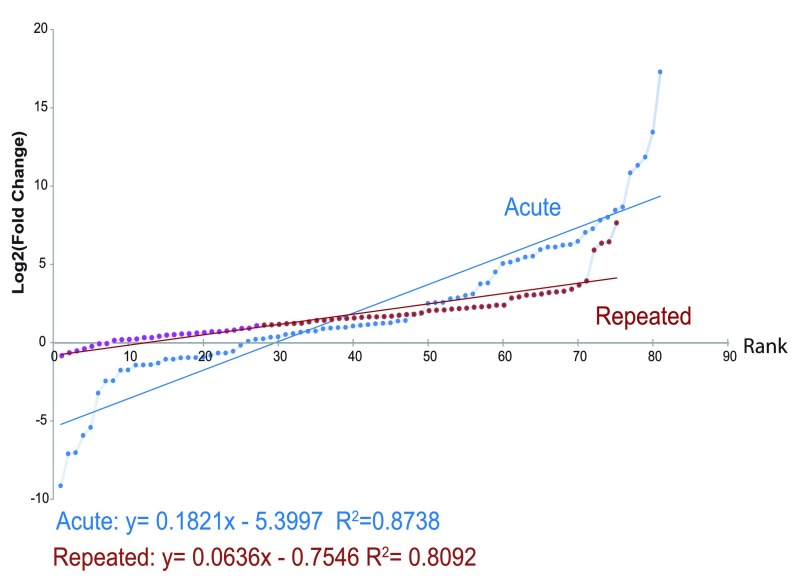
Ranked mean fold change of inflammatory cytokine mRNA transcripts 24 hours after acute (n=4) and repeated bladder inflammation (n=3), as compared to naive data, showing the increased up-regulation present following acute inflammation as compared with repeated inflammation. Following significance of microarray analysis, only inflammatory mediators showing up-regulation were significant (q=0%).

***3.1.2 Repeated bladder inflammation.*** A total of 75/92 cytokine transcripts were analysed for magnitude and significance of transcript up-regulation following repeated bladder inflammation. 16 markers (CCL1, CCL3, CCL25, CCL26, CCL28, CXCL3, IL-2, IL-3, IL-4, IL-9, IL-13, IL-19, IL-20, IL-27, C5, and IFNγ) were excluded from the repeated inflammation model due to high cycle time (>38 overall mean). One housekeeping gene (18s) was excluded as it was significantly up regulated. FC was therefore calculated normalized to the remaining housekeeping genes (HPRT, ACTB, and GAPDH). Figure 2 shows fold change rank for all cytokines analysed. The top 10 mRNA transcripts up-regulated following repeated bladder inflammation with turpentine are shown in Table 3. Using SAM, we identified 4 mRNAs that were significantly different in turpentine compared to naive (FC >1.5, Δ = 1.61, FDR=0%). Of these, 3 were classified as chemokines (CCL7, CCL12, and CXCL17, and 1 as cytokine (IL-1β), as seen in Table 4.

***3.1.3 Comparison of Inflammatory Mediator Up-regulation Following Acute and Repeated Bladder Inflammation.*** From a possible list of 92 transcripts, 73 were detected in both models. Table 5 shows the mean rank, standard deviation, and super rank value (SRV) for each transcript examined. Of these IL-1β was significantly up regulated in both (FDR q=0%). Two other chemokines, CCL7 (acute: 272.74 FC; repeated: 7.15 FC), and CCL12 (acute: 17.96 FC; repeated: 7.15 FC), were also significantly up-regulated in both models (FDR q=0%).

**Table 5.** Super rank values (SRV), consolidating cytokine up-regulation following acute and repeated bladder inflammation with turpentine.

SRV	Gene	Mean Rank	SD
1	Nos2	3	1.41
2	CXCL2	5	0.71
2	Prok2	5	7.07
4	IL-1β	8	2.83
5	CCL12	9.5	4.24
6	IL-1α	13.5	7.07
7	Bdnf	15	8.49
8	C3	18.5	4.95
9	IL-35 / Ebi3	19	4.95
10	Ereg	19.5	10.6

## 3.2 Open field behaviour

**Figure 3.**
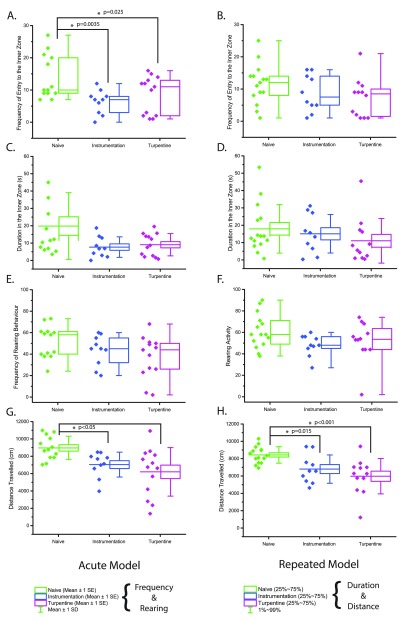
Open field behaviour 24hrs after acute ( **A**, **C**, **E**, **G**; n=9–13) or repeated ( **B**, **D**, **F**, **H**; 10–16) bladder inflammation. **A**/ **E** - Frequency of entry to the inner zone, **B**/ **F** - duration in the inner zone **(s)**, **C**/ **G** - rearing frequency, **D**/ **H** - total distance travelled **(cm)**.

***3.2.1 Acute bladder inflammation.*** Thigmotactic behaviour was observed in animals from both instrumentation and turpentine groups. Animals in both these groups entered the inner zone less frequently compared to naïve (1-way ANOVA, p=0.009). Turpentine animals entered the inner zone 8.6 times (SD 5.84, p=0.025), whereas the instrumentation group averaged 6.3 entries (SD 3.68, p=0.0035), both significant compared to the naïve value of 13.9 entries (SD 6.86) as shown in Figure 3A.

Duration in the inner zone was not significantly different between groups (Kruskal-Wallis 1-way ANOVA, p=0.192). The median time spent in the inner zone was 11.52s (6.68–29.20), 6.80s (2.88–12.96), and 8.64s (2.56–14.32) for naïve, instrumentation, and turpentine groups respectively ( Figure 3C). Rearing behaviour was not significantly different between groups (1-way ANOVA, p=0.112), with mean rear counts of 51.31 (14.96), 43.20 (14.08), and 37.23 (19.70) for naïve, instrumentation, and turpentine groups respectively ( Figure 3E).

Distance travelled was reduced in both instrumentation (7411.62cm IQR 6584.73–7970.77) and turpentine (6744.29cm IQR 3843.16–8192.87) groups when compared to naïve (Dunn’s *post-hoc* test p<0.05, 8771.06cm IQR 7860.85–10,188.78; Figure 3G).

***3.2.2 Repeated Bladder Inflammation.*** No significant effect of group was observed on inner zone frequency (p=0.185) or duration (p=0.288), with naïve entering the inner zone an average of 11.5 (6.22) times with a duration of 13.76s (9.6–23.7), instrumentation averaging 8.7 (5.7) entries with a median duration of 14.48s (6.6–27.2), and turpentine entering the inner zone 7.25 (6.0) times, with a median duration of 6.96s (1.76–16.72) ( Figure 3B and D). No difference was seen in rearing activity (p=0.638), with mean rear counts of 56.9 (13.9), 48.6 (7.7), and 52.22 (21.67) for naïve, instrumentation, and turpentine groups respectively ( Figure 3F).

A decrease in distance travelled was seen in both instrumentation (p=0.015, 6797.29cm SD 1637.10) and turpentine (p=0.00026, 5974.04 cm SD 2039.67) groups compared to naïve (8432.59cm SD 953.15; overall 1-way ANOVA p<0.001, Figure 3H).

## 3.3 c-Fos immunoreactivity in the central amygdala in response to open field exposure

**Figure 4.**
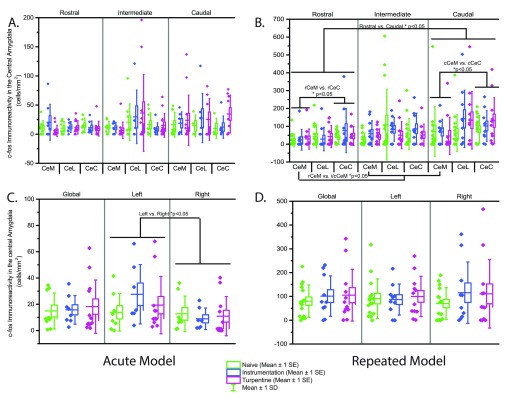
c-Fos immunoreactivity in the central amygdala in response to open field exposure 24hrs after acute ( **A**, **C**; n=8–13) or repeated ( **D**, **F**; n=7–15) bladder inflammation. **A**/ **D** - Global CeA (central amygdala) c-Fos immunoreactivity, **B**/ **E** - right CeA, **C**/ **F** - left CeA.

***3.3.1 Acute Bladder Inflammation.*** Higher levels of c-Fos immunoreactivity were seen in the left hemisphere (p=0.0031), localised to the CeM (p=0.032). No other differences in c-Fos immunoreactivity were observed in the central amygdala, and there was no effect of group, as shown in Figure 4, with full c-Fos density data in Table 6.

***3.3.2 Repeated Bladder Inflammation.*** The CeC showed higher levels of f-cos immunoreactivity compared to the CeM (p=0.00485).

A rostro-caudal gradient was seen with significantly higher levels of activation observed in the caudal regions (p=0.016). Significant differences were also observed within levels, with the rostral CeL (p=0.013) and caudal CeC (p=0.001) showing higher levels when compared to the CeM. c-Fos immunoreactivity in the CeM exhibited a rostro-caudal gradient, with the rostral region containing fewer positive cells compared to the intermediate (p<0.001) and caudal regions (p=0.002). There was no effect of group on c-Fos immunoreactivity ( Figure 4 and Table 6).

**Table 6.** c-Fos immunoreactivity in the central amygdala in response to open field exposure 24hrs after bladder inflammation. Data presented as mean (95% confidence interval). Acute: n=8–13; Repeated: n=7–15. Data shown as mean (SD).

		Acute			Repeated		
		(n)	Mean	SD	(n)	Mean	SD
Global	Naïve	9	14.90	13.68	15	80.07	67.84
	Instrumentation	8	15.78	10.87	12	109.35	106.23
	Turpentine	13	17.07	19.84	10	96.32	80.11
CeM	Naïve	9	12.46	11.56	15	38.57	40.36
	Instrumentation	8	16.43	15.03	11	69.85	71.48
	Turpentine	13	11.94	15.28	10	63.82	61.90
CeL	Naïve	9	18.37	17.14	15	102.51	118.49
	Instrumentation	8	18.77	17.53	12	128.13	127.28
	Turpentine	13	24.95	35.61	9	106.05	105.16
CeC	Naïve	9	15.39	14.41	15	85.11	63.44
	Instrumentation	8	15.23	12.13	12	125.96	146.11
	Turpentine	13	14.76	15.78	10	110.56	120.35
Rostral	Naïve	9	13.24	10.04	15	35.68	38.87
	Instrumentation	8	17.71	19.50	12	62.51	62.65
	Turpentine	13	9.45	10.72	10	55.13	74.87
rCeM	Naïve	9	10.90	12.31	15	21.03	36.25
	Instrumentation	8	20.55	30.80	12	28.87	56.66
	Turpentine	11	6.34	9.29	10	24.07	58.06
rCeL	Naïve	9	10.28	11.44	15	43.34	64.35
	Instrumentation	7	12.30	9.29	12	42.54	62.00
	Turpentine	13	9.93	11.28	10	28.58	65.83
rCeC	Naïve	9	15.25	12.31	15	36.05	36.41
	Instrumentation	8	9.79	11.35	12	45.95	53.22
	Turpentine	13	9.28	12.69	10	75.68	123.36
Intermediate	Naïve	9	15.29	16.00	15	76.56	102.99
	Instrumentation	8	13.05	11.92	12	59.38	62.55
	Turpentine	13	18.86	27.82	10	75.83	69.03
iCeM	Naïve	9	11.97	9.85	15	25.62	35.61
	Instrumentation	7	11.13	9.02	12	40.17	45.61
	Turpentine	13	6.68	14.15	10	58.73	72.41
iCeL	Naïve	9	20.63	31.44	15	109.55	198.56
	Instrumentation	6	29.79	45.97	12	50.20	76.14
	Turpentine	11	39.75	68.04	10	82.66	66.41
iCeC	Naïve	9	16.64	20.33	15	56.19	87.06
	Instrumentation	8	9.06	10.30	12	50.95	61.50
	Turpentine	12	16.44	20.75	10	85.96	87.11
Caudal	Naïve	9	16.18	17.90	15	107.27	136.63
	Instrumentation	8	16.56	22.56	12	120.59	140.43
	Turpentine	13	22.90	29.71	10	114.58	102.95
cCeM	Naïve	9	16.32	21.38	15	69.99	139.71
	Instrumentation	6	18.82	19.43	12	46.16	103.93
	Turpentine	13	23.79	43.28	10	68.25	71.35
cCeL	Naïve	4	14.39	18.66	15	34.54	41.57
	Instrumentation	7	27.68	42.25	12	189.22	283.47
	Turpentine	9	22.45	34.54	10	143.61	51.92
cCeC	Naïve	4	9.90	17.47	15	44.86	66.93
	Instrumentation	7	12.17	19.04	12	138.74	156.49
	Turpentine	10	31.07	32.48	10	136.03	81.07

## 3.4 Correlations

***3.4.1 Acute Bladder Inflammation.*** Overall, there was a positive correlation between rearing activity and c-Fos immunoreactivity in the rostral CeC (Pearson’s ρ=0.44, p=0.01).

Looking at correlations within experimental groups, the naïve group showed the highest levels of correlation, with rearing activity positively correlated with CeC (Overall, right hemisphere, and rostral level; ρ=0.73–80, p<0.05) and CeM (caudal; ρ=0.72, p=0.03). Distance travelled was also positively correlated with caudal c-Fos immunoreactivity in the left central amygdala (ρ=0.8, p=0.02).

In the instrumentation group, there was a positive correlation between duration in the inner zone and c-Fos immunoreactivity in the intermediate CeL (ρ=0.76, p=0.05). No correlations were observed in the turpentine group.

See Table 7 and Table S2 for full details of all correlations.

**Table 7.** Summary of Pearson co-efficient correlations between behavioural outcomes and c-Fos immunoreactivity in the central amygdala following both acute and repeated bladder inflammation. Correlations conducted at overall and group level. Bold* denotes p>0.05. ABBREVIATIONS: N, Naïve; I, Instrumental; T, Turpentine; CeM, medial central amygdala; CeL, lateral central amygdala; CeC, capsular central amygdala.

	Acute												Repeated											
	Distance			Frequency			Duration			Rearing			Distance			Frequency			Duration			Rearing		
Global	0.30			0.21			0.05			0.14			0.03			0.21			0.17			-0.40		
	0.23	0.21	0.09	0.31	0.39	0.18	0.36	0.51	-0.07	0.01	-0.30	-0.58	0.51	0.12	0.35	0.29	-0.15	0.22	-0.07	-0.14	0.27	0.65	-0.18	0.18
CeM	0.20			0.09			-0.10			0.22			0.10			0.23			0.16			-0.51		
	0.49	0.08	0.34	0.38	0.13	0.43	0.23	0.27	0.19	-0.02	-0.45	-0.71*	0.28	-0.10	0.25	-0.10	0.12	0.12	-0.36	-0.12	0.08	0.58	0.08	**0.27**
CeL	0.28			0.20			0.19			-0.04			0.14			0.25			0.20			-0.32		
	0.30	0.43	0.11	0.39	0.39	0.12	0.45	0.35	-0.11	-0.05	-0.50	-0.55	0.56	0.27	0.38	0.53	-0.24	0.28	0.45	0.01	0.39	0.21	-0.22	0.01
CeC	0.24			0.18			-0.05			0.19			0.02			0.19			0.13			-0.33		
	0.17	0.19	0.10	0.28	0.46	0.16	0.31	0.56	-0.09	0.22	-0.11	-0.52	0.40	-0.29	0.23	0.17	-0.34	0.12	-0.27	-0.52	0.13	0.73	-0.44	0.29
Left	0.26			0.20			0.09			0.07			0.13			0.27			0.26			-0.31		
	0.44	0.18	0.07	0.44	0.14	0.22	0.52*	0.22	-0.02	-0.08	-0.51	-0.53	0.50	0.14	0.39	0.42	-0.14	0.32	0.16*	-0.06	0.35	0.49	-0.13	0.06
Right	0.32			0.18			-0.04			0.23			-0.04			0.17			0.12			-0.41		
	-0.07	0.20	0.06	0.09	0.49	0.19	0.09*	0.60	-0.05	0.14	-0.18	-0.62	0.33	0.09	0.24	-0.10	-0.10	0.08	-0.43*	-0.28	0.11	0.62	-0.14	0.29
Rostral	0.06			0.01			-0.10			0.18			0.10			0.25			0.26			-0.25		
	0.66	0.03	0.34	0.38	0.37	0.32	0.41*	0.53	0.12	0.20	-0.39	-0.44	0.23	-0.21	-0.06	-0.08	-0.01	-0.13	-0.34*	-0.21	-0.09	0.53	-0.08	0.32
Intermediate	0.28			0.24			0.11			0.09			0.26			0.31			0.41			0.16		
	0.41	**0.25**	0.23	0.45	0.15	0.16	0.53*	0.22	0.27	0.10	-0.15	0.29	0.44	**0.13**	0.31	0.35	-0.11	0.18	-0.05*	0.21	0.28	0.66	-0.18	0.14
Caudal	0.31			0.19			0.05			0.09			0.05			0.28			0.30			-0.30		
	0.12	0.11	0.09	0.52	0.21	0.09	0.68*	0.31	-0.15	-0.06	-0.43	-0.53	0.66	0.29	0.46	0.39	-0.16	0.35	0.08*	-0.15	0.33	0.60	-0.10	0.08
	N	I	T	N	I	T	N	I	T	N	I	T	N	I	T	N	I	T	N	I	T	N	I	T
	Overall			Overall			Overall			Overall			Overall			Overall			Overall			Overall		

***3.4.2 Repeated Bladder Inflammation.*** Overall, there were correlations between frequency, duration, and rearing and c-Fos immunoreactivity in the central amygdala. Frequency was positively correlated with CeL immunoreactivity in the left hemisphere (ρ=0.35, p=0.03), and CeM immunoreactivity in the right hemisphere (ρ=0.35, p=0.04) and caudal level (ρ=0.39, p=0.02). Duration in the inner zone was positively correlated with rostral CeL (ρ=0.33, p=0.05), caudal CeM (ρ=0.37, p=0.02), and c-Fos immunoreactivity in all intermediate divisions except the right hemisphere (ρ=0.34–0.41, p<0.05). Rearing was negatively correlated with CeM (overall, left, right, and rostral; ρ=-0.43- -0.67, p<0.05), right overall (ρ=-0.41, p=0.05), right CeL (ρ=-0.46, p=0.02), and caudal CeC (ρ=-0.52, p=0.01).

Within the naïve group, there were positive correlations between distance travelled and rostral c-Fos immunoreactivity (overall, left, and CeL; ρ=0.58–0.67, p<0.02). Frequency in the inner zone was positively correlated with caudal (overall, CeM; ρ=0.52–0.61, p<0.05) and rostral CeL c-Fos immunoreactivity (ρ=0.58, p=0.02). Duration in the inner zone was positively correlated with CeC (left, intermediate; ρ=0.59, p=0.02), CeL (left, rostral; 0.53–0.62, p<0.04), caudal CeM (ρ=0.66, p=0.01), left hemisphere (overall, intermediate, caudal; ρ=0.52–0.56, p<0.05), and intermediate (ρ=0.53, p=0.04). No correlations were seen with rearing behaviour.

In the instrumentation group, distance travelled was positively correlated with left CeL (ρ=0.71, p=0.02), and rearing was negatively correlated with left rostral c-Fos immunoreactivity (ρ=-0.93, p=0.02).

In the turpentine group, duration in the inner zone was correlated with intermediate CeM c-Fos immunoreactivity (ρ=0.64, p=0.02). Rearing behaviour was negatively correlated with c-Fos immunoreactivity in the CeM (overall, right, caudal; ρ=-0.71- -0.90, p<0.03), and in the right hemisphere (rostral, CeL; ρ=-0.76- -0.78, p<0.02).

See Table 7 and Table S2 for full details of all correlations.

## 4. Discussion

Both acute and repeated bladder inflammation up-regulate inflammatory mediator expression, most notably IL-1β, CCL-7, and CCL-12. The fold change was higher in the acute model compared to the repeated, suggestive of a habituation effect when an animal is repeatedly exposed to an inflammatory stimulus. Thigmotactic alterations were observed in both instrumentation and turpentine groups following acute but not repeated bladder inflammation, although reductions in locomotor activity were observed in both models. Investigations into amygdalar c-Fos immunoreactivity following bladder inflammation were inconclusive.

Numerous studies investigating cytokine expression profiles across experimental models of inflammatory pain show results similar to those reported here. IL-6, IL-10, and TNFα are highly expressed in normal bladder tissue ( Pokrywczynska *et al.*, 2013), and have pro-inflammatory actions ( de Oliveira *et al.*, 2011). Additionally, in a study examining cytokine expression following UVB irradiation, five markers also seen in our model of acute bladder inflammation were up-regulated: CCL-4, CCL-7, IL-1β, IL-24, and iNos/Nos2, of which IL-1β and CCL-7 were also seen in our repeated model ( Dawes *et al.*, 2011). In an acute model of pelvic pain (experimental prostatitis) there was increased expression of both CCL7 and CCL12, and seven other chemokines also seen in acute bladder inflammation (CCL2, CCL3, CCL4, CCL6, CXCL2, CXCL3, and XCL1), suggesting commonality in inflammatory mediators up-regulated in pelvic inflammatory models ( Quick *et al.*, 2012). Considering the effects of systemic cytokines on behaviour, peripheral IL-1β is associated with decreased activity in the open field, whether administered exogenously ( Campbell *et al.*, 2010; Song *et al.*, 2005), or endogenously up-regulated in response to inflammatory stimuli such as lipopolysaccharide (LPS) ( Swiergiel & Dunn, 2007) and intra-plantar carrageenan ( Wen *et al.*, 2014). In a study combining peripheral and systemic effects of cytokines with behaviour and c-Fos immunoreactivity in the amygdala, IL-1β, TNFα, and IL-6 reduced activity, with TNFα and IL-1β also associated with increased c-Fos immunoreactivity in the central amygdala ( Skelly *et al.*, 2013).

Open field behaviour was reduced in both turpentine and instrumentation groups following acute and repeated bladder inflammation. Distance travelled was decreased in both instrumentation and turpentine groups following acute and repeated bladder inflammation. Exaggerated thigmotactic behaviour (reduced frequency and duration of inner zone visits) was observed only in the acute model, but in both turpentine and instrumentation groups, suggesting catheterisation and instillation of vehicle (olive oil) could be capable of creating an aversive state. Notably, a similar trend was also seen in the repeated, although this did not reach significance due to high levels of variation. Previous studies examining cyclophosphamide (CYP)-induced cystitis revealed mixed effects on behaviour: male mice showed reduced rearing activity but increased time active in the open field four hours after acute injection ( Olivar & Laird, 1999), however a recent study found no difference distance travelled in the open field following CYP treatment in female rats ( Coelho *et al.*, 2014). Other models of visceral inflammation show similarly mixed effects: trinitrobenzenesulfonic acid (TNBS)-induced acute pancreatitis increased immobility in male mice three weeks post-insult ( Cattaruzza *et al.*, 2013), but this effect was not seen in the cerulein-induced model of pancreatitis ( Michalski *et al.*, 2007). Chronic iodoacetamide (IAA)-induced gastritis reduced inner zone activity in female but not male rats ( Luo *et al.*, 2013), whereas acute intra-colonic instillation of mustard oil is reported to either reduce open field locomotor activity ( Maia *et al.*, 2006), or have no effect ( Leite *et al.*, 2012). A similar model of persistent colonic inflammation (deoxycholic acid/DCA) also failed to detect differences in distance travelled in the open field ( Traub *et al.*, 2008). However, intra-plantar CFA has been shown to induce thigmotactic behaviour without reducing distance travelled in the open field, up to 30 days after the original insult, suggesting the effects of acute inflammation can persist ( Parent *et al.*, 2012). Our data supports the idea that inflammation is capable of changing behaviour twenty-four hours after inflammation, but that this effect is not specific to introduction of an irritant into the bladder, and there is an element of habituation, as suggested by the reduced effects observed in the repeated model.

The presence of behavioural alteration in both surgical groups suggests the instillation procedure in itself is enough to alter behaviour. The volume instilled (0.5ml) is within the physiological range, accounting for diurnal variation (Herrera & Meredith, 2010), suggesting the pressure generated would be insufficient to initiate a noxious stretch response. Therefore, olive oil and/or catheterisation are capable of influencing open field behaviour. Anti-nociceptive effects of intra-peritoneal olive oil injection have been shown ( Eidi *et al.*, 2012), and there are numerous studies investigating its purported anti-inflammatory effects in relation to the Mediterranean diet (for review see Lucas *et al.*, 2011), suggesting olive oil may have a protective rather than inflammatory effect. On the other hand, catheterisation is known as a risk factor for cystitis ( Dayts, 2014; Tenke *et al.*, 2014) and pelvic pain ( Nazarko, 2014), suggesting further investigation is required into mechanisms involved in the behavioural alterations we saw in our instrumentation groups.

In the current study, we failed to detect an effect of instrumentation or turpentine on c-Fos immunoreactivity. Increased activity was seen in the lateral (CeL; rostral) and capsular (CeC; caudal) when compared with the medial central amygdala (CeM). The CeL and CeC often considered together as they both receive input from the spinal cord, and brainstem, as well as signals from higher regions such as the cortex, via the thalamus ( Neugebauer *et al.*, 2004). We noted significantly higher levels of activation in the caudal regions of the central amygdala, and although the biological significance of this is not known, it has been previously observed in a model of CYP-induced cystitis ( Bon *et al.*, 1998). No significant effects were seen in the acute model, suggesting the link between open field outcomes and central amygdala activation may be time-dependent, however the lack of robust correlations between behaviour and c-Fos immunoreactivity in this model could also be indicative of reduced involvement of the CeA in the inflammatory-mediated behavioural alterations we observed. Another factor to consider is the higher levels of immunoreactivity seen in the repeated model compared to the acute model. This is observed across all experimental groups, suggesting an environmental effect associated with differences in the housing of the animals.

Other studies involving inflammatory models have shown associated increases in central amygdala activation: c-Fos immunoreactivity in the central amygdala was increased 60 minutes after acute intra-peritoneal injection of the gastric hormone CCK (cholecystokinin) ( Rinaman, 2003), and intra-plantar formalin was associated with an increased c-Fos immunoreactivity detectable for up-to 2 ( Rouwette *et al.*, 2011). However, our data is not directly comparable with these studies as the acute timescale considered was considerably shorter than the twenty-four hours we studied. Furthermore, our experiment was designed to investigate whether recent visceral inflammation alters central amygdala activity in response to the open field, as opposed to c-Fos in response to noxious stimuli alone.

A study looking at blood flow and proliferation (cell number and volume) found these measures increased in the central amygdala following spared nerve injury, without a concomitant alteration in elevated plus maze or open field behaviour ( Gonçalves *et al.*, 2008). Nonetheless, thigmotaxis has been observed in association with increased central amygdala activity in peripheral nerve trauma ( Burke *et al.*, 2013), and post-operative incisional models ( Li *et al.*, 2010), emphasising the complexity of central amygdala involvement the generation of thigmotactic behavioural alterations.

We found a significant negative correlations between CeM c-Fos immunoreactivity and rearing behaviour in the repeated turpentine group in particular, suggesting rearing behaviour may be suppressed by CeM activity, although we failed to detect significant alterations in rearing. c-Fos immunoreactivity in the CeM was generally lower than that seen in the CeL/C, as would be expected when considering the CeL and CeC are involved in modulation of incoming noxious signals, whereas the CeM is thought to be more involved in initiation of the behavioural response. Investigations into the direct effect of experimental pain states on amygdala activation have shown up-regulation in the central amygdala, ( Dai *et al.*, 1993; Hayashi *et al.*, 2009; de Lange *et al.*, 2005; Lehner *et al.*, 2006; Rea *et al.*, 2011; Rouwette *et al.*, 2012; Yamashiro *et al.*, 1998; Yang *et al.*, 2010) but none have investigated differences between nuclear subdivisions.

High levels of variability were observed across all data sets, suggesting innate behavioural variation. Numerous studies have shown evidence for a divergent response to stress, and typically note the presence of two behavioural phenotypes – those that have low activity and high neophobia (low activity/high “anxiety”), and those that show higher levels of activity, and low levels of neophobia (high activity/low “anxiety”) ( Koolhaas, 2008; Koolhaas *et al.*, 2010; Mällo *et al.*, 2007; Kabbaj *et al.*, 2000; Landgraf & Wigger, 2002). We noted the presence of distinctive behavioural phenotypes in all groups including naive, characterised by low or high thigmotaxis, and are developing techniques to investigate these differences further – ideally, animals would be behaviourally phenotypes prior to inflammation in order to determine the effects of trait characteristics on state responses. A previous study has shown differential c-Fos expression in animals with differing behavioural phenotypes, namely up-regulation of central c-Fos expression in low activity/high “anxiety” rats ( Salomé *et al.*, 2004). However, although the rats used in our study were the same strain (Wistar, Charles River, UK), those in the Salome study were bred for high and low anxiogenic phenotype in the elevated plus maze (F12), which would likely magnify underlying neurochemical differences present in the original strain. Additionally, the duration of open field exposure was 30 minutes, as compared to the 15 minutes in our study, and it is likely that this would affect the c-Fos activation profile as the animal habituates to its environment.

To fully elucidate the c-Fos response to open field exposure, and determine whether the increase in caudal activation we observed has biological significance, further experiments comparing animals with and without exposure to the open field are required. Investigating cytokine profiles in more individuals would allow further correlation between behavioural outcomes and physiological pathology, and could be particularly instructive in understanding the effect seen in the instrumentation groups. Studies have shown behavioural responses to systemic IL-1β are variable ( Pertsov *et al.*, 2009), and also that acute responses can vary with oestrus cycle in female rats ( Avitsur *et al.*, 1995), suggesting investigations taking this into account may increase the robustness of this study, although a literature survey conducted in 2014 found similar variability between male and female animals ( Prendergast *et al.*, 2014). Furthermore, there are potential refinements that could be applied to this model to facilitate testing behaviour at earlier time points, including reducing the duration of instillation and therefore anaesthesia required by using a more specific and/or potent irritant. Finally, testing of behavioural phenotype prior to treatment allocation would allow more detailed study of whether and how behavioural phenotype modulates responses to visceral inflammation.

In summary, bladder inflammation is associated with a robust up-regulation of peripheral cytokines implicated in pain, inflammation, and behavioural depression, and acutely with increased thigmotaxis, not specific to inflammation. The data on neural correlates are inconclusive, but as increased variation in these outcomes is observed, further studies are required to elucidate mechanisms responsible.

## Data availability

*Figshare:* Cytokine q-RT-PCR, c-Fos immunoreactivity and open field behaviour data in rats following bladder inflammation doi: 10.6084/m9.figshare.1394861 ( Morland *et al.*, 2015).

## Figures and Tables

**Table S1.** Cytokine qRT-PCR Array Card Targets.

Chemokines	Cytokines	Growth Factors	Enzymes	Complement System	Other	Housekeeping Genes
CCL1	M- CSF1	Areg	Alox5	C3	Aif1	18S
CCL2	GM- CSF2	Artn	Alox15	C5	Edn1	Actb
CCL3	G- CSF3	Bdnf	Lta4h		PROK2	Gapdh
CCL4	Ifng	Btc	Nos2			Hprt1
CCL5	IL-1α	Ereg	Ptges			
CCL6	IL-1β	Fgf7	Ptgs2			
CCL7	IL-2	Hbegf				
CCL9	IL-3	Kitlg				
CCL11	IL-4	Ngf				
CCL12	IL-5	Nrg1				
CCL17	IL-6	Nrg1 (exon j)				
CCL19	IL-7					
CCL20	IL-9					
CCL21b	IL-10					
CCL22	IL-11					
CCL24	IL-12a					
CCL25	IL-12b					
CCL26	IL-13					
CCL27	IL-14 / Txlna					
CCL28	IL-15					
CX3CL1	IL-16					
CXCL1	IL-17 / CTLA-8	
CXCL2	IL-18					
CXCL3	IL-19	
CXCL4 / Pf4	IL-20					
CXCL6	IL-21	
CXCL7 / Ppbp	IL-23a					
CXCL9	IL-24					
CXCL10	IL-27					
CXCL12	IL-33					
CXCL13	IL-35 / Ebi3					
CXCL14	Lif					
CXCL16	Mif					
CXCL17	Tnf					
XCL1						

**Table S2.** Full Pearson’s correlations between behavioural outcomes and c-Fos immunoreactivity in the central amygdala. Bold denotes p<0.05.

		Acute												Repeated											
		Distance			Frequency			Duration			Rearing			Distance			Frequency			Duration			Rearing		
Global	Overall	0.30			0.21			0.05			0.14			0.03			0.21			0.17			-0.40		
		0.23	0.21	0.09	0.31	0.39	0.18	0.36	0.51	-0.07	0.01	-0.30	-0.58	0.51	0.12	0.35	0.29	-0.15	0.22	-0.07	-0.14	0.27	0.65	-0.18	0.18
	CeM	0.20			0.09			-0.10			0.22			0.10			0.23			0.16			-0.51		
		0.49	0.08	0.34	0.38	0.13	0.43	0.23	0.27	0.19	-0.02	-0.45	**-0.71**	0.28	-0.10	0.25	-0.10	0.12	0.12	-0.36	-0.12	0.08	0.58	0.08	**0.27**
	CeL	0.28			0.20			0.19			-0.04			0.14			0.25			0.20			-0.32		
		0.30	0.43	0.11	0.39	0.39	0.12	0.45	0.35	-0.11	-0.05	-0.50	-0.55	0.56	0.27	0.38	0.53	-0.24	0.28	0.45	0.01	0.39	0.21	-0.22	0.01
	CeC	0.24			0.18			-0.05			0.19			0.02			0.19			0.13			-0.33		
		0.17	0.19	0.10	0.28	0.46	0.16	0.31	0.56	-0.09	0.22	-0.11	-0.52	0.40	-0.29	0.23	0.17	-0.34	0.12	-0.27	-0.52	0.13	0.73	-0.44	0.29
Left	Overall	0.26			0.20			0.09			0.07			0.13			0.27			0.26			-0.31		
		0.44	0.18	0.07	0.44	0.14	0.22	**0.52**	0.22	-0.02	-0.08	-0.51	-0.53	0.50	0.14	0.39	0.42	-0.14	0.32	**0.16**	-0.06	0.35	0.49	-0.13	0.06
	CeM	0.11			0.01			-0.16			0.18			0.08			0.13			0.16			-0.49		
		0.50	0.12	0.32	-0.02	0.16	0.49	0.07	0.32	0.26	-0.20	-0.18	-0.64	0.28	-0.05	0.34	0.15	0.16	0.17	-0.24	-0.07	0.05	0.66	0.14	0.19
	CeL	0.26			0.24			0.29			-0.07			0.29			0.35			0.29			-0.16		
		0.47	**0.71**	0.21	0.50	0.42	0.15	**0.53**	0.18	-0.02	-0.09	-0.46	-0.33	0.60	**0.41**	0.26	0.55	-0.13	0.25	**0.57**	0.22	0.37	0.13	-0.07	-0.14
	CeC	0.13			0.02			-0.09			0.04			0.04			0.25			0.21			-0.33		
		0.29	0.08	-0.05	0.50	0.00	0.17	**0.59**	0.06	-0.12	0.09	0.25	-0.63	0.12	-0.21	0.23	-0.07	-0.31	0.17	**-0.26**	-0.43	0.22	0.48	-0.42	0.26
Right	Overall	0.32			0.18			-0.04			0.23			-0.04			0.17			0.12			-0.41		
		-0.07	0.20	0.06	0.09	0.49	0.19	**0.09**	0.60	-0.05	0.14	-0.18	-0.62	0.33	0.09	0.24	-0.10	-0.10	0.08	**-0.43**	-0.28	0.11	0.62	-0.14	0.29
	CeM	0.25			0.17			0.05			0.18			0.11			0.35			0.19			-0.43		
		0.29	0.03	0.28	0.49	0.22	0.42	0.25	0.30	0.18	0.09	-0.66	-0.84	0.23	-0.34	0.06	-0.33	-0.15	0.02	-0.40	-0.34	0.08	0.38	-0.27	0.24
	CeL	0.10			-0.01			-0.10			-0.01			-0.03			0.11			0.10			-0.46		
		-0.08	**0.22**	-0.21	0.11	0.21	0.07	**0.22**	0.22	-0.10	0.06	-0.41	-0.78	-0.13	**-0.09**	0.30	-0.07	-0.33	0.11	**-0.37**	-0.37	0.15	0.23	-0.39	0.16
	CeC	0.31			0.31			0.00			0.32			0.00			0.15			0.09			-0.29		
		-0.01	0.19	0.13	-0.05	0.52	0.17	**-0.11**	0.62	-0.04	0.29	-0.13	-0.44	0.52	-0.34	0.22	0.32	-0.18	0.06	**-0.22**	-0.43	0.02	0.76	-0.18	0.30
Rostral	Overall	0.06			0.01			-0.10			0.18			0.10			0.25			0.26			-0.25		
		0.66	0.03	0.34	0.38	0.37	0.32	**0.41**	0.53	0.12	0.20	-0.39	-0.44	0.23	-0.21	-0.06	-0.08	-0.01	-0.13	**-0.34**	-0.21	-0.09	0.53	-0.08	0.32
	Left	-0.16			-0.22			-0.24			-0.01			0.07			0.15			0.23			-0.06		
		0.58	-0.09	0.36	0.10	0.15	0.45	0.23	0.32	0.36	0.08	-0.93	-0.15	-0.16	-0.23	-0.25	-0.15	-0.13	-0.17	-0.32	-0.32	-0.15	0.39	-0.19	0.22
	Right	0.32			0.30			0.10			0.34			0.07			0.28			0.21			-0.40		
		0.40	**0.08**	0.06	0.49	0.39	0.14	**0.43**	0.45	-0.10	0.26	-0.28	-0.76	0.35	**0.03**	0.02	-0.03	0.48	-0.09	**-0.34**	0.22	-0.05	0.52	0.40	0.30
	CeM	0.00			-0.06			-0.09			0.13			0.06			0.14			0.12			-0.67		
		0.44	-0.23	0.25	-0.05	-0.06	0.54	**0.08**	0.07	0.26	-0.34	-0.78	-0.90	0.23	-0.02	-0.21	-0.28	0.28	-0.12	**-0.18**	0.01	-0.01	0.12	0.26	-0.16
	CeL	0.13			0.02			-0.17			0.12			0.26			0.31			0.33			0.26		
		0.67	0.33	0.10	0.58	0.39	-0.09	**0.62**	0.34	-0.05	0.18	-0.77	0.37	-0.20	0.20	-0.01	-0.08	-0.22	-0.14	**-0.48**	-0.04	-0.10	0.39	-0.23	0.24
	CeC	0.29			0.30			0.01			0.44			0.10			0.20			0.25			0.12		
		0.50	0.10	0.29	0.25	0.46	0.02	0.21	0.58	0.05	0.49	-0.18	0.31	0.36	0.14	0.00	0.13	0.49	-0.10	-0.37	0.00	-0.10	0.80	0.40	0.32
Intermediate	Overall	0.28			0.24			0.11			0.09			0.26			0.31			0.41			0.16		
		0.41	**0.25**	0.23	0.45	0.15	0.16	**0.53**	0.22	0.27	0.10	-0.15	0.29	0.44	**0.13**	0.31	0.35	-0.11	0.18	**-0.05**	0.21	0.28	0.66	-0.18	0.14
	Left	0.22			0.22			0.13			0.03			0.26			0.31			0.41			0.04		
		0.42	0.24	0.24	0.46	0.12	0.06	**0.56**	0.25	0.13	-0.19	0.08	0.39	0.34	0.22	0.23	0.41	0.05	0.13	**0.09**	0.40	0.22	0.50	-0.01	-0.09
	Right	0.19			0.10			-0.03			0.15			0.03			0.11			0.19			-0.05		
		0.20	0.09	0.04	0.05	0.20	0.20	**0.07**	0.23	0.38	-0.13	-0.58	0.05	0.27	-0.27	0.18	-0.22	-0.42	0.07	**-0.43**	-0.49	0.12	0.52	-0.48	0.28
	CeM	0.20			0.14			-0.01			0.32			0.06			0.24			0.38			0.07		
		0.49	0.28	0.02	0.24	0.34	0.44	0.35	0.44	0.64	-0.28	-0.58	0.50	0.09	-0.45	0.04	-0.26	-0.18	-0.02	-0.39	-0.57	-0.01	0.62	-0.16	0.36
	CeL	0.16			0.11			0.08			-0.07			0.29			0.32			0.34			-0.07		
		0.36	**0.36**	0.21	0.39	0.11	0.15	**0.43**	0.12	0.15	-0.19	-0.06	-0.19	0.44	**0.33**	0.25	0.49	0.16	0.11	**0.17**	0.76	0.25	0.47	0.05	-0.25
	CeC	0.35			0.30			0.08			0.19			0.10			0.23			0.36			0.02		
		0.26	0.09	0.11	0.46	-0.02	0.16	**0.59**	0.07	0.26	-0.02	0.22	-0.01	0.38	0.29	0.15	0.19	-0.10	-0.02	**-0.18**	-0.10	0.02	0.61	-0.07	0.22
Caudal	Overall	0.31			0.19			0.05			0.09			0.05			0.28			0.30			-0.30		
		0.12	0.11	0.09	0.52	0.21	0.09	**0.68**	0.31	-0.15	-0.06	-0.43	-0.53	0.66	0.29	0.46	0.39	-0.16	0.35	**0.08**	-0.15	0.33	0.60	-0.10	0.08
	Left	0.34			0.28			0.16			0.05			0.16			0.25			0.23			-0.32		
		0.24	0.37	0.12	0.41	0.05	0.13	0.56	-0.09	-0.14	-0.21	0.03	-0.51	0.80	0.36	0.46	0.64	-0.22	0.38	0.39	-0.16	0.36	0.34	-0.10	0.08
	Right	0.08			-0.08			-0.19			-0.09			-0.10			0.00			-0.03			-0.37		
		-0.28	**0.00**	0.08	-0.11	0.13	0.11	**-0.02**	0.27	-0.10	-0.15	-0.65	-0.47	0.21	**0.24**	0.23	0.04	-0.15	0.10	**-0.34**	-0.23	0.04	0.65	-0.14	-0.24
	CeM	0.28			0.11			-0.07			0.11			0.19			0.39			0.37			-0.34		
		0.31	-0.03	0.22	0.61	-0.02	0.23	**0.66**	0.09	-0.04	-0.01	-0.27	-0.88	0.46	0.16	0.41	0.20	-0.13	0.19	**-0.22**	0.07	0.07	0.72	-0.23	0.09
	CeL	0.25			0.03			0.06			-0.03			-0.09			-0.02			-0.06			-0.34		
		-0.16	0.20	0.07	-0.10	0.30	0.02	**-0.12**	0.36	-0.17	-0.06	-0.56	-0.44	0.68	0.19	0.44	0.12	-0.28	0.41	**0.66**	-0.35	0.43	-0.42	-0.16	0.04
	CeC	0.18			0.09			-0.10			0.02			-0.10			0.05			0.04			-0.52		
		-0.16	0.05	0.07	-0.25	0.13	0.43	-0.11	0.21	0.22	-0.40	-0.33	-0.59	0.10	0.34	0.42	-0.44	-0.15	0.39	-0.46	-0.24	0.42	0.78	-0.06	-0.09

## Supplementary materials

**ARRIVE Checklist**.
