# Supplementary material for: Short-term effect of acute and repeated urinary bladder inflammation on thigmotactic behaviour in the laboratory rat
Source: F1000Res. 2015 May 8;4:109. [Version 1] doi: 10.12688/f1000research.6255.1 (PMC4850861; doi:10.12688/f1000research.6255.1)
Supplement: Peer review history

# Peer review: Referee response for version 1

**DOI:** 10.5256/f1000research.6710.r8593

**Published:** 2015-05-22

**Authors:** Hugo Leite-Almeida

**Affiliations:** 1 Life and Health Sciences Research Institute (ICVS), School of Health Sciences, University of Minho, Braga, Portugal

In the manuscript entitled “Short-term effect of acute and repeated urinary bladder inflammation on thigmotactic behaviour in the laboratory rat” authors report their observations on the effects of acute and repeated bladder inflammation in open-field associated behavioral parameters like locomotion, thigmotaxis and rearings. In addition, the expression of inflammatory mediators in the bladder was analyzed by RT-qPCR and c-fos expression was quantified in the amygdala. Authors observed that turpentine driven inflammation in the bladder resulted in the up-regulation of inflammatory mediators particularly following acute challenge; these observations were not controlled for the effect of catheterization. Turpentine inflammation was associated with a decreased number of entries in the central area of the open-field area; in this case authors controlled for the effect of catheterization alone and observed that this group displayed a similar phenotype. Again, acutely challenged subjects were more affected. C-fos expression in the amygdala following open-field exposure was inconclusive.

The manuscript is well-written and is in many aspects exemplar, adhering to the best practices in data reporting (sample size calculation, inclusion/exclusion criteria for experimental subjects, randomization, among others). Also, authors made available an interesting set of raw data permitting reanalysis to anyone interested. Finally, authors stress the importance of having alternative measures in pain models that go beyond sensory testing.

  

There are however some aspects that need clarification:

- Authors preformed an exhaustive characterization of inflammation-associated transcripts expressed in the bladder; it is not clear what was the gain of this approach in the context presented by the authors in the introduction to the study; after all the model involved trauma (associated with the catheterization) and the injection of turpentine, therefore inflammation in the bladder was not an unexpected outcome. On the contrary, I would see some advantages if authors would have treated animals with anti-inflammatory drugs or, more importantly, if the strategy was applied in CNS tissues (namely in the amygdala); indeed a number of studies have associated the up-regulation of inflammatory mediators with anxiety-like behaviors.  
- Instrumentation and turpentine animals present a robust decrease in the distance travelled in the open-field most probably as a result of inflammation-driven sickness behavior. Because open-field readouts depend on animal’s ability to move, other behavioral parameters can be indirectly affected. Have authors controlled for this? Have other measures be taken like animals’ weight, grooming, body temperature, etc? These would be particularly important in the repeated model.
- c-fos density values presented for naïve animals present an increase over 5 fold between acute and repeated models (in the global analysis; similar increases can be found in the segmented analysis); apart from open-field exposure naïve animals should by definition be equal. My concern is that such effect can mask biologically relevant variations of c-fos expression (e.g. ceiling effect).

I have read this submission. I believe that I have an appropriate level of expertise to confirm that it is of an acceptable scientific standard.

# Peer review: Referee response for version 1

**DOI:** 10.5256/f1000research.6710.r8597

**Published:** 2015-05-19

**Authors:** Nicholas Andrews

**Affiliations:** 1 Department of Neurology, Children's Hospital Boston, Harvard Medical School, Boston, MA, USA

This is very detailed description of an interesting study attempting to bring together behaviour, anatomy and neurochemical responses. The methods are very clearly described. I have only a few minor points for the authors to consider.

Introduction

The case is made for the amygdala in relation to pathways involved in pain relay but not made for combining with open field. The open field arguably elicits thygmotaxis through a neophobic and spatial effects which might relate more closely to the hippocampus. A little more in the introduction discussing the known relationship between the amygdala and open field behaviour would be helpful to the reader and place the choice of test in greater context.

Results

Can the reduction in frequency of entries into the centre be explained by a general reduction in motor activity? If this is accounted for in an ANCOVA does the reduced time in the centre become more or less pronounced?

Was velocity altered between the groups? Might also reflect wariness?

Discussion

Is it possible that the variation of responses in the open field is due to the lack of clear salient stimuli for causing the thygmotaxis? A more discrete training stimulus as evident in conditioned fear-based paradigms may give less variability. Have the authors considered using other such tests, especially in light of the strong body of evidence relating conditioned fear to amygdala function?

I have read this submission. I believe that I have an appropriate level of expertise to confirm that it is of an acceptable scientific standard.

# Peer review: Referee response for version 1

**DOI:** 10.5256/f1000research.6710.r8596

**Published:** 2015-05-19

**Authors:** Gordon Munro

**Affiliations:** 1 Department of Pharmacology, NeuroSearch A/S, Ballerup, Denmark

The manuscript submitted by Morland *et al. *has assessed whether rats with acute or more prolonged bladder inflammation induced by turpentine oil exhibit thigmotactic behaviour as assessed in the open field paradigm. In addition, regulation of inflammatory mediators within the bladder and c-Fos immunoreactivity within the central amygdala was assessed.

The authors provide a clearly defined hypothesis which they address appropriately with a number of the methodologies at hand. In the absence of a direct method to assess pain-like behaviour in the various groups I like that the authors assessed inflammatory mediator profiles using a well established methodology previously utilized in other experimental pain models (e.g. UV burn) where pain-like behaviours were measured directly. The manuscript is well written. The data have been analysed using appropriate statistical tests and the results are extensively described. Although the data ultimately reveal that instrumentation rather than inflammation *per se* can sufficiently induce thigmotactic behaviour this should not detract from the value of this work. Rather, the extensive description of study design, powering, randomization, blinding, exclusions and availability of their data for the scrutiny of their peers makes this work in many ways a first, and an extremely valuable addition to the field in my opinion. This said I have a number of issues with some of the included work as described in more detail below.

**Major points**

1. The value of the current work would have been considerably enhanced if the experimental design had included a separate arm in which vehicle treated rats were treated with an anxiolytic dose of e.g. diazepam, or a motor impairing dose of an opiate like morphine with corresponding plasma samples obtained for purposes of drug exposure. Despite the extensive detail provided within the Methods section I find it extremely difficult to judge the sensitivity of their open field paradigm without the availability of this kind of information.
2. The inclusion of the c-Fos data in the context of the current manuscript is to be blunt, both confusing and distracting. This is not because the data are inconclusive *per se, *in that no differences in c-Fos immunoreactivity were noted between instrumentation or turpentine treated groups. I would have expected that the authors might have seen a more robust pattern/magnitude of c-Fos immunoreactivity if rats in these 2 groups had been sacrificed at 90 minutes after induction of the procedure rather than the following day following open field performance. Whilst careful consideration and extensive reporting of experimental design was provided in relation to performing behavioural analysis, I just can’t see that the same is the case for the purposes of assessing ‘Amygdala activation’. Incidentally, without knowing precisely the function and phenotype of the c-Fos positive cells included in their analysis, I would suggest that this term is misleading – I think the authors might be aware of this as indicated at the bottom of page 14. The authors specify that sequential sections were obtained from each rat. I would presume that in a number of cases depending upon the exact plane of the sections that the same positive neurones were measured more than once. Accordingly, with section numbers ranging from 1-7 per rat as indicated in Table 2 this must introduce some form of bias between treatments. This issue goes against the grain of the rigorous experimental designs employed for purposes of behavioural analyses As such, I would strongly recommend that the authors remove all c-Fos related method and results from the manuscript. Alternatively, they could provide videocapture of all sections used in their analyses and provide this for open review to see if others can replicate their finding

**Minor points**

1. When performing c-Fos immuno were all sections processed within the same experimental run? If not, were assays blocked appropriately to contain representative samples from each of the treatment groups?
2. Following on from the above, a generalized positive control group (with a background in neuroendocrinology I would have included a group of rats treated with eg hypertonic saline and measured c-Fos expression within specific hypothalamic nuclei such as the SON or PVN) would have provided a general measure of assay sensitivity.
3. Can the authors comment on whether all rats were tested during similar stages of the oestrus cycle? Does this matter?

I have read this submission. I believe that I have an appropriate level of expertise to confirm that it is of an acceptable scientific standard.
